# Glycogen Synthase Kinase 3β Enhances Hepatitis C Virus Replication by Supporting miR-122

**DOI:** 10.3389/fmicb.2018.02949

**Published:** 2018-11-27

**Authors:** Maged Saleh, Sabrina Rüschenbaum, Christoph Welsch, Stefan Zeuzem, Darius Moradpour, Jérôme Gouttenoire, Christian M. Lange

**Affiliations:** ^1^Department of Internal Medicine 1, University Hospital Frankfurt, Frankfurt, Germany; ^2^Division of Gastroenterology and Hepatology, Centre Hospitalier Universitaire Vaudois, University of Lausanne, Lausanne, Switzerland

**Keywords:** GSK3α, GSK3β, hepatitis E virus, host-targeting antivirals, insulin resistance, miR-122

## Abstract

Hepatitis C virus (HCV) infection is associated with alterations in host lipid and insulin signaling cascades, which are partially explained by a dependence of the HCV life cycle on key molecules in these metabolic pathways. Yet, little is known on the role in the HCV life cycle of glycogen synthase kinase 3 (GSK3), one of the most important kinases in cellular metabolism. Therefore, the impact of GSK3 on the HCV life cycle was assessed in human hepatoma cell lines harboring subgenomic genotype 1b and 2a replicons or producing cell culture-derived HCV genotype 2a by exposure to synthetic GSK3 inhibitors, GSK3 gene silencing, overexpression of GSK3 constructs and immunofluorescence analyses. In addition, the role of GSK3 in hepatitis E virus (HEV) replication was investigated to assess virus specificity of the observed findings. We found that both inhibition of GSK3 function by synthetic inhibitors as well as silencing of GSK3β gene expression resulted in a decrease of HCV replication and infectious particle production, whereas silencing of the GSK3α isoform had no relevant effect on the HCV life cycle. Conversely, overexpression of GSK3β resulted in enhanced HCV replication. In contrast, GSK3β had no effect on replication of subgenomic HEV replicon. The pro-viral effect of GSK3β on HCV replication was mediated by supporting expression of microRNA-122 (miR-122), a micro-RNA which is mandatory for wild-type HCV replication, as GSK3 inhibitors suppressed miR-122 levels and as inhibitors of GSK3 had no antiviral effect on a miR-122-independent HCV mutant. In conclusion, we have identified GSK3β is a novel host factor supporting HCV replication by maintaining high levels of hepatic miR-122 expression.

## Introduction

Hepatitis C virus is a member of the *Flaviviridae* family which has a positive-sense single-stranded RNA genome (Lange et al., 2014b). The HCV genome encodes the viral structural and non-structural proteins, which are required for the HCV life cycle (Lange et al., 2014b). In addition to HCV proteins, a number of host factors have been discovered on which HCV critically depends (Lange et al., 2014b). Though most of these host factors are cellular proteins, a liver-specific micro-RNA (miR-122) has been identified which is mandatory for HCV replication and which is (at least partially) responsible for the hepatotropism of this virus (Lange et al., 2014b).

Chronic infection with HCV is not only a major cause of liver cirrhosis and hepatocellular carcinoma, but also associated with metabolic traits including insulin resistance and dyslipidemia (Kawaguchi et al., 2004; Shintani et al., 2004; Bose et al., 2012). While the effects of HCV infection on glucose metabolism are clearly documented *in vivo* and *in vitro*, the benefit of these alterations for the virus remains largely unclear (Petta et al., 2010; Negro, 2014), although insulin resistance was identified as a negative predictor of outcome of interferon-based therapies (Romero-Gómez et al., 2005; Dai et al., 2009; Grasso et al., 2009; Moucari et al., 2009; Khattab et al., 2010; Fattovich et al., 2011).

Gglycogen synthase kinase 3 is a serine/threonine protein kinase that exists in two isoforms, GSK3α and GSK3β, which are encoded by two different genes. Approximately 100 substrates of GSK3 have been proposed, highlighting a central role for GSK3 in numerous cellular processes such as proliferation, migration, apoptosis, immune modulation and - importantly - glucose metabolism (Jope et al., 2007). Of note, substrates of GSK3α and GSK3β are overlapping only partially. The activity of GSK3 is modulated by inhibitory phosphorylation at Ser 21 for GSK3α and Ser 9 for GSK3β by upstream kinases, where the phosphorylated N-terminal tail acts as a pseudosubstrate that prevents incoming substrates from entering the catalytic center (Cohen and Frame, 2001).

The importance of GSK3 is further highlighted by its role in the pathogenesis of relevant diseases including diabetes, cancer and inflammation (Beurel et al., 2015). Accordingly, several GSK3 inhibitors are in preclinical and clinical development, for example for the treatment of Alzheimer’s disease or diabetes (del Ser et al., 2013; Lovestone et al., 2015). Of note, GSK3 has also been identified as a host factor required for replication of influenza and SARS viruses (Wu et al., 2009; König et al., 2010). Yet, the role of GSK3 in the HCV life cycle remains to be characterized.

In view of the close relationship between HCV infection and metabolic alterations, the present study aimed at investigating a possible role of GSK3 in the HCV life cycle.

## Materials and Methods

### Cell Culture, Subgenomic Replicons, Cell Culture-Derived HCV, Plasmids

Huh-7.5 human hepatoma cell line was provided by Charles M. Rice (The Rockefeller University, New York, NY) and cultured in DMEM (Life Technologies, Carlsbad, CA) containing 10% heat-inactivated FCS. HCV subgenomic replicon construct pCon1/SG-Neo(I)/AflII (Con1 strain, genotype 1b) (Blight et al., 2000) was provided by Charles M. Rice. Full-length HCV construct pFK-JFH1J6C-846_dg (Jc1 virus, genotype 2a) (Pietschmann et al., 2006) was provided by Ralf Bartenschlager (University of Heidelberg, Germany). HCV U3 virus, a genotype 2a full-length strain resistant to miR-122 inhibition was provided by Jens Bukh (University of Copenhagen, Denmark) (Li et al., 2011). Huh-7.5 cells were electroporated with *in vitro* transcribed full-length HCV RNA and 72 h later the virus infectivity in the supernatant was assessed by foci forming unit (ffu) determination using anti-HCV core monoclonal antibody (mAb) C7-50 (Moradpour et al., 1996), as described (Zhong et al., 2005). The S10-3 cell line harboring a HEV genotype 3 replicon derived from Kernow-C1 p6 strain (provided by Suzanne U. Emerson (National Institutes of Health, MD) was used as previously described (Dao Thi et al., 2016). Sofosbuvir (Alsachim SAS, Illkirch-Graffenstaden, France), HCV NS5B inhibitor, was resolved in DMSO and used at a final concentration of 1 μM. GSK3 inhibitors lithium chloride (LiCl) and CHIR99021 (Sigma-Aldrich, St. Louis, MO, United States) were resolved in sterile water and DMSO, respectively. Cytotoxicity was assessed using the WST-1 assay (Sigma-Aldrich).

Haemagglutinin-tagged GSK3β wild-type plasmid (HA-GSK3β wt pcDNA3) was a gift from Jim Woodgett (Lunenfeld-Tanenbaum Research Institute, Mount Sinai Hospital, Toronto, ON, Canada) (Addgene plasmid #14753) (He et al., 1995). Transfection of GSK3 constructs for overexpression was performed with a final DNA concentration of 0.75 μg/ml using X-tremeGENE HP DNA transfection reagent (Roche Diagnostics, Mannheim, Germany).

### Immunoblot Analysis

Immunoblotting was performed as described previously (Lange et al., 2014a). Antibodies against total GSK3 α/β (5676S), phosphorylated-GSK3 α/β (Ser9/21) (9331S), and β-catenin (9562S) were purchased from Cell Signaling Technologies (Danvers, MA, United States). Mouse MAb 9E10 against HCV NS5A was provided by Charles M. Rice (Lindenbach, 2005) and mAb 12B7 against HCV NS5B was described earlier (Moradpour et al., 2002). ECL donkey HRP-conjugated anti-rabbit (NA934V) and HRP-conjugated anti-mouse (sc-2031) secondary antibodies were from GE Healthcare (Little Chalfont, United Kingdom) and Santa Cruz Biotechnology (Dallas, TX, United States), respectively.

### Reverse Transcription and Quantitative PCR

Total RNA extraction was done using RNeasy Mini Kits and miRNeasy Mini Kit for mRNA and micro-RNAs, respectively (Qiagen, Hilden, Germany). Reverse transcription of mRNA and micro-RNA was performed using the PrimeScript RT reagent kit (Takara Bio, Otsu, Japan) and the TaqMan MicroRNA Reverse Transcription Kit (Applied Biosystems, Foster City, CA, United States), respectively.

For real-time PCR, StepOnePlus Real-Time PCR system (Applied Biosystems) was used. For HCV and GAPDH, experiments were carried out using TaqMan Gene Expression master mix (Applied Biosystems). For micro-RNAs, TaqMan Universal Master Mix II (Applied Biosystems) was used for quantification of the miRNA cDNAs. The following primers were used for HCV amplification: forward, 5′-ACGCAGAAAGCGTCTAGCCAT-3′, reverse, 5′-TACTCA CCGGTTCCGCAGA-3′, HCV TaqMan probe, 5′-TCCTGGAGGCTGCACGACACTCA-3′. TaqMan Gene Expression Assay containing the pre-designed primers and TaqMan probe from Applied Biosystems was used for GAPDH. For miR-122 and miR-16, pre-designed primers (Assay ID no. 2245 and 2420, respectively) from Thermo Fisher Scientific were employed. For HEV, the procedure and primers have been previously described (Dao Thi et al., 2016). Results were calculated using the 2^-ΔΔCT^ method and shown as fold change compared to control.

### Gene Silencing

Gene silencing was performed as described previously (Lange et al., 2014a) using predesigned siRNAs from Life Technologies. Single siRNAs for GSK3α (s6236 and s6237) and β (s6239 and s6240) as well as a non-targeting control siRNA were transfected at a final concentration of 5 nM using Lipofectamine RNAiMAX (Life Technologies).

## Results

### Inhibition of GSK3 Kinase Activity Suppresses HCV Replication

**FIGURE 1:**
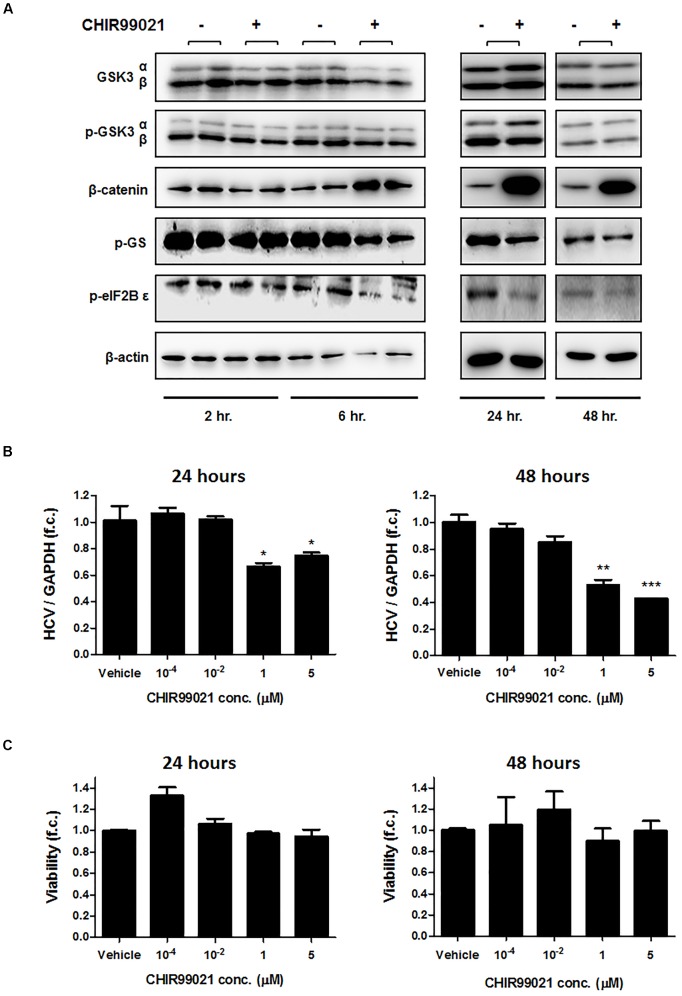
GSK3 inhibition suppresses HCV replication. **(A)** Confirmation of GSK3 inhibition by CHIR99021. Immunoblot analysis was carried out for total and phosphorylated-GSK3α/β (Ser 9/21), β-catenin, phosphorylated glycogen synthase (GS), eukaryotic initiation factor 2B𝜀, and β-actin after treatment of Huh-7.5 cells harboring Con1 replicon with 5 μM CHIR99021 for 2, 6, 24 and 48 h, as indicated. **(B)** Suppression of subgenomic HCV replication by GSK3 inhibition. Huh-7.5 cells harboring Con1 replicon were treated with different concentrations of CHIR99021 for 24 and 48 h. HCV RNA levels normalized to GAPDH mRNA are expressed relative to untreated cells. **(C)** Assessment of GSK3 inhibitors’ cytotoxicity. WST-1 cytotoxicity assay was carried out for cells stimulated for 24 and 48 h with different concentrations of CHIR99021 at seeding densities of 10,000 and 15,000 cells per well in 96-well plates. Absorbance readings (A_450_ – A_690_ nm) of treated cells were compared to negative control cells treated with the dimethyl sulfoxide vehicle. Data are presented as mean ± SEM of six independent experiments. Asterisks denote statistically significant differences (^∗^*P*-value ≤ 0.05; ^∗∗^*P*-value ≤ 0.005; ^∗∗∗^*P*-value ≤ 0.0005).

CHIR99021 is a highly selective small molecule inhibitor of GSK3α and GSK3β that inhibits GSK3 by competition with ATP in the ATP-binding site of the kinase (Ring et al., 2003; Meijer et al., 2004). To prove efficacy of GSK3 inhibition by CHIR99021, immunoblot analyses of β-catenin, phosphorylated glycogen synthase (GS), and phosphorylated eIF2B 𝜀 as downstream targets of GSK3 were performed. As shown in Figure 1A, β-catenin was upregulated and phosphorylated GS and eIF2B 𝜀 were downregulated by CHIR99021, as expected.

**FIGURE 2:**
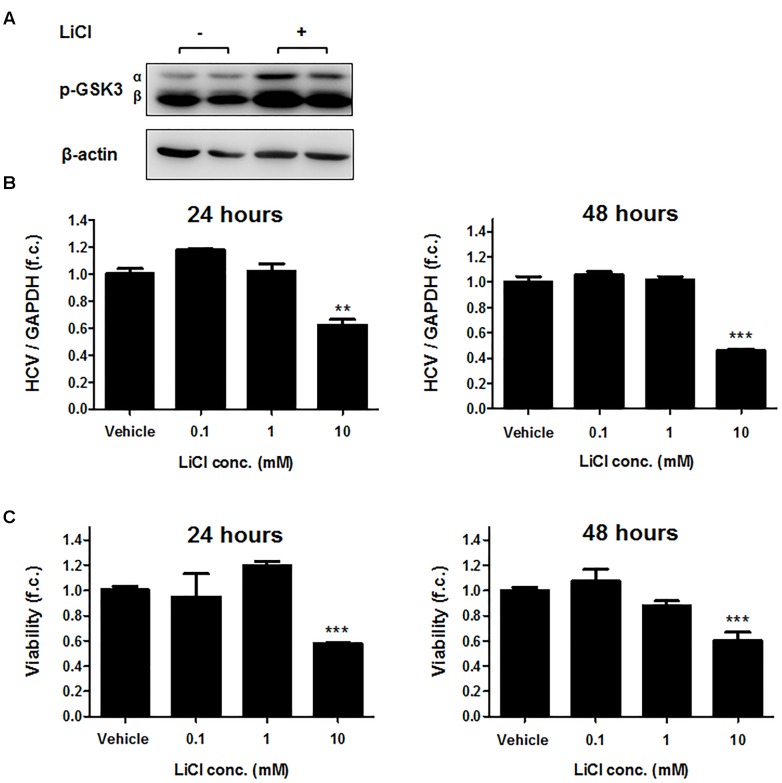
Lithium chloride suppresses HCV replication. **(A)** Immunoblot analysis was carried out for phosphorylated-GSK3α/β (Ser 9/21) after stimulation of Huh-7.5 cells harboring Con1 replicon with 25 mM LiCl for 2 h. β-actin was detected as a loading control. **(B)** Suppression of subgenomic HCV replication by LiCl. Huh-7.5 cells harboring Con1 replicon were stimulated with LiCl for 24 or 48 h at the indicated concentrations. HCV RNA levels normalized to GAPDH mRNA are expressed relative to untreated cells. **(C)** Assessment of lithium chloride-induced cytotoxicity. WST-1 cytotoxicity assay was carried out for cells stimulated for 24 and 48 h with LiCl at the indicated concentrations. Absorbance readings (A_450_ – A_690_ nm) of treated cells were compared to negative control cells treated with the dimethyl sulfoxide vehicle. Data are presented as mean ± SEM of six independent experiments. (^∗∗^*P*-value ≤ 0.005; ^∗∗∗^, *P*-value ≤ 0.0005).

We next exposed Huh-7.5 cells harboring the subgenomic replicon (Con1 strain) to different concentrations of CHIR99021 for 24 and 48 h and quantified HCV RNA by PCR. As shown in Figure 1B, treatment with CHIR99021 led to an inhibition of HCV RNA replication. Of note, no relevant effect on cell viability was observed as a consequence of GSK3 inhibition, as assessed by WST-1 assay (Figure 1C). Comparable results were observed for treatment of Huh-7.5 cells harboring the subgenomic Con1 replicon with lithium chloride, another inhibitor of GSK3 (Beurel et al., 2015) (Figure 2).

**FIGURE 3:**
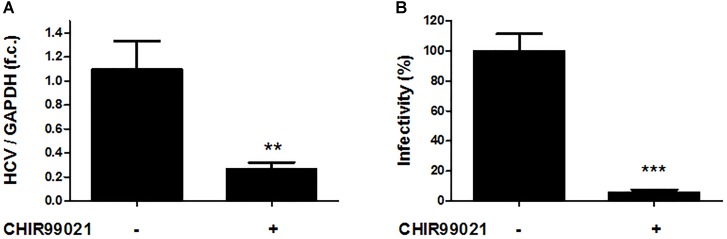
GSK3β inhibition suppresses full-length HCV replication as well as production of infectious HCV particles. **(A)** Inhibition of infectious HCV clone Jc1 replication by GSK3 inhibition. Huh-7.5 cells electroporated with HCV Jc1 full-length RNA were treated with 5 μM CHIR99021 for 72 h post-transfection. HCV RNA levels normalized to GAPDH mRNA are expressed relative to untreated cells. Data are presented as mean ± SEM of two experiments performed in triplicate. f.c., fold change. **(B)** Suppression of HCV infectious particles production by GSK3 inhibition. Virus infectivity was determined by foci forming assay in supernatants from Huh-7.5 cells electroporated with HCV Jc1 RNA and cultured 48 h in the presence of 5 μM CHIR99021. Results were shown as infectivity level normalized to control samples cultured in presence of 0.1% dimethyl sulfoxide vehicle. Asterisks denote statistically significant differences (^∗∗^*P*-value ≤ 0.005; ^∗∗∗^*P*-value ≤ 0.0005).

Next, Huh-7.5 cells electroporated with full-length HCV genotype 2a Jc1 RNA were treated with CHIR99021 for 72 h to assess the effects of GSK3 inhibition on infectious HCV. As shown in Figure 3A, GSK3 inhibition resulted in a profound inhibition of replication of the infectious HCV clone, as well as of particle production (Figure 3B).

### GSK3β, but Not GSK3α, Is Required for the HCV Life Cycle

**FIGURE 4:**
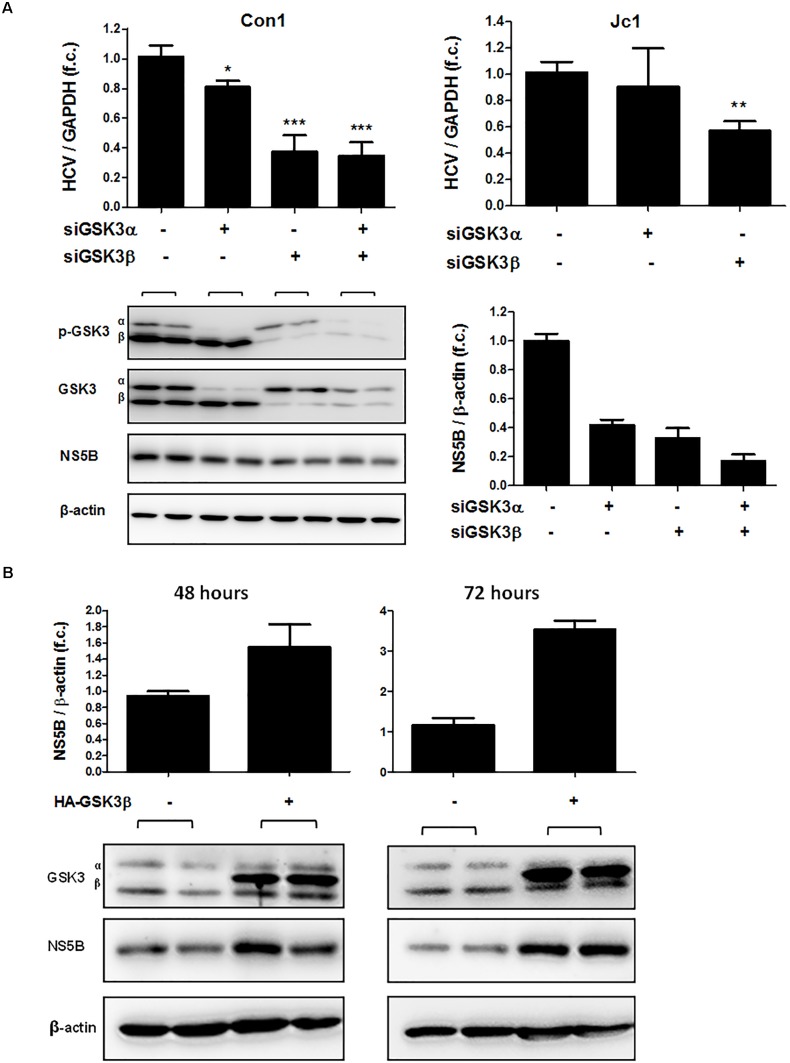
GSK3β is required for HCV replication, not GSK3α. **(A)** GSK3β silencing suppresses HCV replication. Huh-7.5 cells harboring subgenomic Con1 replicon (left panel) or replicating the full-length Jc1 clone were transfected with siRNAs of GSK3α (siRNA #6236), β (siRNA #6239) or both for 72 h. HCV RNA levels normalized to GAPDH mRNA are expressed relative to untreated cells. Data are presented as mean ± SEM of two experiments performed in triplicate. Asterisks denote statistically significant differences (^∗^*P*-value ≤ 0.05; ^∗∗^*P*-value ≤ 0.005; ^∗∗∗^*P*-value ≤ 0.0005). Immunoblot analysis was carried out for GSK3α/β to confirm gene silencing efficiency, and for HCV NS5B to assess suppression of HCV replication on the protein level (A, lower panel). β-actin was detected as a loading control. NS5B signal intensities were normalized to β-actin for quantification and were presented as mean ± SEM of signal intensities of duplicate bands. **(B)** GSK3β overexpression promotes HCV expression. Huh-7.5 cells harboring Con1 replicon were transfected with pcDNA3 plasmid expressing HA-tagged wild-type GSK3β (HA-GSK3β) for 48 and 72 h. Immunoblot analysis was carried out for GSK3α/β to confirm transfection efficiency. The overexpression was confirmed by visualizing HA-GSK3β distinct bands with a slightly higher molecular weight than that of the endogenous GSK3β. HCV NS5B protein was detected to assess HCV replication level and β-actin was detected as a loading control. NS5B signal intensities were normalized to β-actin for quantification and were presented as mean ± SEM of signal intensities of duplicate bands.

To assess whether both GSK3 isoforms are required for HCV replication, we silenced GSK3α and / or GSK3β gene expression by transfecting siRNAs and quantified HCV RNA in Huh-7.5 cells harboring HCV subgenomic replicon (Con1) or infectious HCV (Jc1). As shown in Figure 4A (upper panel), silencing of GSK3β gene expression resulted in a substantial decrease of HCV RNA levels of both the subgenomic replicon and the full-length HCV construct, whereas silencing of GSK3α gene expression had only a minor effect on HCV replication. Comparable results were observed for a second set of siRNAs directed against GSK3α or GSK3β (Supplementary Figure 1). The suppressive effect of GSK3β gene silencing on HCV replication was further confirmed on the protein level by quantifying HCV NS5B protein (Figure 4A lower panel). Furthermore, the enhancing effect of GSK3β on HCV RNA replication was confirmed by overexpression of a functional GSK3β construct, which increased HCV RNA replication (Figure 4B).

### The Pro-viral Effects of GSK3β Are Specific for HCV

**FIGURE 5:**
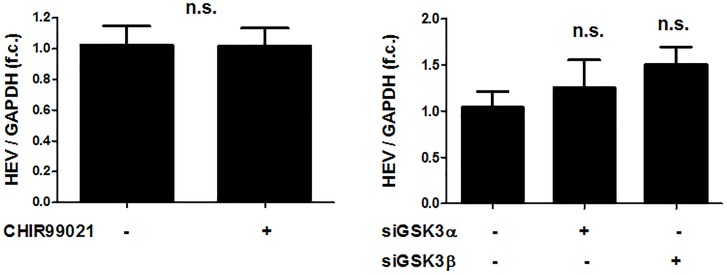
The pro-viral effects of GSKβ are limited to HCV in comparison to HEV. No effects of GSK3 inhibition on HEV replication. Huh-7-derived cells (S10-3) harboring a selectable HEV replicon construct (HEV Kernow-C1 strain) were treated with 5 μM CHIR99021 for 48 h or transfected with siRNAs to silence GSK3α and β gene expression for 72 h. HEV RNA levels normalized to GAPDH mRNA are expressed relative to negative control siRNA-transfected cells. Data are presented as mean ± SEM of two experiments performed in triplicate and compared by Wilcoxon M Whitney *U*-test (n.s., not significant) with the control group.

To test whether the pro-viral effects of GSK3β are specific for HCV, we assessed the impact of GSK3 inhibition on replication of HEV, another hepatotropic positive-strand RNA virus. As shown in Figure 5, neither silencing of GSK3α and / or GSK3β gene expression nor treatment with the GSK3 inhibitor CHIR99021 had a relevant effect on HEV RNA levels, suggesting that the pro-viral effect of GSK3β is specific to HCV.

### GSK3β Inhibition Suppresses HCV Replication Through Downregulation of microRNA-122

**FIGURE 6:**
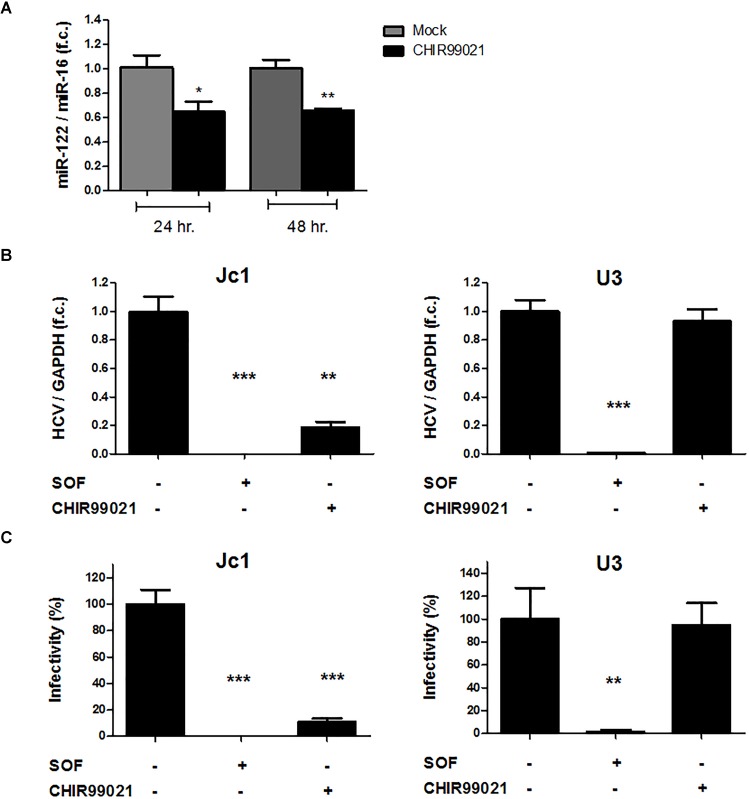
GSK3 inhibition suppresses HCV by reduces miR-122 expression level. **(A)** GSK3 inhibition reduces miR-122 expression level. Huh-7.5 cells harboring HCV Con1 replicon were stimulated with 5 μM CHIR99021 for 24 and 48 h. MiR-122 expression level was normalized to miR-16 level as endogenous control. **(B)** GSK3 inhibition has no effects on miR-122-resistant HCV. Huh-7.5 cells, pre-treated for 24 h with 0.1% dimethyl sulfoxide (DMSO), 1 μM sofosbuvir (SOF) or 5 μM CHIR99021, were infected with either Jc1 or miR-122-resistant U3 virus (MOI = 0.1) and treated in the same conditions for 72 h before harvesting. HCV RNA levels normalized to GAPDH mRNA level are expressed relative to DMSO-control. Data are presented as mean ± SEM of two experiments performed with six samples each. **(C)** GSK3 inhibition has no effects on miR-122-resistant HCV infectious particles production. Infectivity was determined by foci forming units (ffu) assay in cell supernatants harvested 72 h post-infection of Jc1 or U3 RNA and treated with either 1 μM SOF or 5 μM CHIR99021. Results were shown as infectivity level normalized to control samples cultured in presence of 0.1% dimethyl sulfoxide vehicle. For all experiments, data are presented as mean ± SEM of two experiments performed with six samples each. Asterisks denote statistically significant differences (^∗^*P*-value ≤ 0.05; ^∗∗^*P*-value ≤ 0.005; ^∗∗∗^*P*-value ≤ 0.0005).

Given the well-known dependency of HCV on miR-122 (Jopling et al., 2005, 2008; Machlin et al., 2011) and the regulatory circuit between insulin-like growth factor 1 receptor, GSK3β, and miR-122 identified previously (Zeng et al., 2010), we assessed the effect of GSK3 inhibition on miR-122 expression in Huh-7.5 cells harboring a subgenomic HCV replicon. As shown in Figure 6A, treatment with CHIR99021 resulted in downregulation of miR-122 levels, in a similar magnitude compared to HCV replication inhibition. For further validation, Huh-7.5 cells transfected with HCV Jc1 or U3 full-length RNA, the latter being a HCV construct replicating independently from miR-122, were treated with CHIR99021. As shown in Figure 6B, CHIR99021 inhibited the replication of the full-length HCV Jc1 genome, the replication of which depends on the presence of miR-122. In contrast, CHIR99021 had no inhibitory effect on replication, nor entry and particle production, of the HCV U3. In line with these observations, the production of Jc1 infectious viral particles was strongly inhibited by CHIR99021 whereas this inhibitor had no effect on infectious particle production of the U3 virus (Figure 6C), suggesting that GSK3β inhibition mainly impacts HCV RNA replication.

## Discussion

In view of remarkable associations between HCV and metabolic traits, we have investigated the role in the HCV life cycle of GSK3, a key kinase in glucose metabolism and numerous other cellular processes. Our study shows that GSK3β, but not GSK3α, serves as an important host factor of viral RNA replication. The pro-viral effects of GSK3β appeared to be mediated, at least in part, by maintaining hepatocellular levels of the key microRNA miR-122.

MicroRNA-122 is an important microRNA predominantly expressed in hepatocytes, where it accounts for approximately 50–70% of the entire microRNA pool. The high abundancy of miR-122 in liver cells underlines key functions in liver development, growth, differentiation and homeostasis (Bandiera et al., 2015). It is not surprising, therefore, that repression of miR-122 expression promotes the development of hepatocellular carcinoma (HCC). In addition, miR-122 is involved in important metabolic functions of the liver including cholesterol and fatty acid synthesis and iron homeostasis (Esau et al., 2006; Castoldi et al., 2011). A unique feature of the HCV life cycle is its dependence on the presence of miR-122, which stabilizes the HCV genome and promotes viral replication through binding to the 5′-untranslated region (5′-UTR) of the viral genome (Jopling et al., 2005, 2008; Machlin et al., 2011). Given the liver-specific expression of miR-122, this micro-RNA is also considered to be a key determinant of the hepatotropism of HCV. The strong dependence of HCV on miR-122 is evidenced by profound suppression of viral loads by therapeutic silencing of miR-122 *in vivo* (Lanford et al., 2010; Janssen et al., 2013). Given the tumor suppressor function of miR-122, these therapeutic silencing strategies may be associated with an increased risk of HCC development. In addition, a recent study has shown that HCV functionally regulates (sequesters) miR-122 within hepatocytes, which results in a de-repression of miR-122 target genes in the presence of HCV and which may partially explain the oncogenic potential of chronic hepatitis C (Luna et al., 2015). Our finding that specifically GSK3β (and not GSK3α) is required to maintain miR-122 levels in HCV infected hepatocytes adds another facet to the complex reciprocal relationship between HCV, miR-122 and the implications of chronic HCV infection on metabolic and oncogenic traits. Yet, it is a limitation of our study that we cannot completely exclude that targets of GSK3 other than miR-122 are additionally involved in the suppression of HCV replication by GSK3 inhibition. However, it appears plausible that depletion of miR-122 plays a key role in mediating suppression of HCV replication by GSK3-inhibition because miR-122 is one of the most important host factors on which HCV replication critically depends. A further confirmation of this notion is the important finding that the miR-122-independent HCV mutant is resistant to GSK3 inhibition. In addition, GSK3 inhibition had no suppressive effect on replication of HEV, a virus which does – in contrast to HCV – not depend on miR-122.

Our results confirm a study by Zeng *et al.,* which has described a regulatory circuit between GSK3, miR-122 and insulin-like growth-factor 1 receptor in hepatocytes (Zeng et al., 2010). GSK3 is a multifunctional protein with an estimated 100 different substrates, thereby regulating numerous cellular processes and pathways beyond glucose metabolism. Hence, inappropriate GSK3 signaling appears to be involved in diverse diseases such as diabetes mellitus, cancer or Alzheimeŕs disease. Though a number of GSK3 inhibitors are in preclinical and clinical development, there is a concern of serious side effects due to the pleiotropic effects of GSK3. However, some of the most significant undesirable effects of GSK3 inhibition, in particular β-catenin accumulation with potential deleterious carcinogenic effects, develop as a result of combined inhibition of GSK3α and GSK3β (Rayasam et al., 2009). In this regard, the finding that only GSK3β but not GSK3α affects miR-122 levels is important because it may serve as a proof-of-principle that selectively targeting GSK3 isoforms may be a suitable approach to manipulate specific functions of GSK3. Our data further illustrate the need for dissecting the role of GSK3 isoforms separately instead of non-specifically referring to GSK3 inhibition.

In view of the pleiotropic cellular functions of GSK3 it is not surprising that viruses engage this important kinase to complete their life cycle. It has been shown that replication of the SARS coronavirus depends on GSK3-mediated phosphorylation of the nucleocapsid (N) protein which is a necessary process for viral replication (Wu et al., 2009). In addition, it was shown that GSK3β promotes the life cycle of influenza virus through facilitating the viral entry step (König et al., 2010). The here reported role of GSK3β in the HCV life cycle adds another facet to the dependence of viruses on this central kinase. Furthermore, the role of GSK3 in the HCV life cycle may be not restricted to HCV replication, as a recent report has shown that GSK3 supports HCV assembly by its involvement in lipoprotein production (Sarhan et al., 2017). While we did not make this observation in our experimental settings, our data indicate that it likely remains marginal compared to the role of GSK3 in supporting HCV RNA replication. Yet, the importance of these and our findings may rather consist in its implications for the understanding of the pathogenesis of chronic hepatitis C than in the identification of a novel target of antiviral therapy. GSK3 is a downstream target of insulin which is inhibited by insulin receptor signaling (Welsh and Proud, 1993). Reciprocally, GSK3 inhibits pivotal downstream targets of the insulin receptor under nonstimulated conditions, such as glycogen synthase (Woodgett and Cohen, 1984; Roach, 1991) and IRS-1 (Eldar-Finkelman and Krebs, 1997; Liberman and Eldar-Finkelman, 2005). As a result, increased GSK3 activity has been observed in diabetic tissues (Eldar-Finkelman et al., 1999; Nikoulina et al., 2000) and inhibition of GSK3 activity improves insulin resistance (Cline et al., 2002; Henriksen et al., 2003; Nikoulina et al., 2002). Consequently, one may speculate that the dependence of HCV on active GSK3 could explain a benefit for HCV of insulin resistance, which is induced by hepatitis C and which had been associated with lower chances of treatment-induced viral clearance in the era of interferon therapy (Romero-Gómez et al., 2005).

## Author Contributions

MS, SR, CW, JG, and CL collected the data. MS, SR, SZ, DM, JG, and CL analyzed the data. All authors have contributed to the manuscript by planning the study, and preparing as well as revising the manuscript.

## Conflict of Interest Statement

CL: speaker fees from Gilead Sciences, AbbVie. Unrestricted research support from Gilead Sciences. The remaining authors declare that the research was conducted in the absence of any commercial or financial relationships that could be construed as a potential conflict of interest.

## Abbreviations

DAAs: direct-acting antivirals
FCS: fetal calf serum
GSK3: glycogen synthase kinase 3
HCV: hepatitis C virus
HEV: hepatitis E virus
IRS-1: insulin receptor substrate-1
miR-122: microRNA-122
PCR: polymerase chain reaction
SARS: severe acute respiratory syndrome
siRNA: small interfering RNA
