# Supplementary material for: Glycogen Synthase Kinase 3β Enhances Hepatitis C Virus Replication by Supporting miR-122
Source: Front Microbiol. 2018 Nov 27;9:2949. doi: 10.3389/fmicb.2018.02949 (PMC6278592; doi:10.3389/fmicb.2018.02949)
Supplement: Supplementary file 1 [file Image_1.pdf]

**SI Information – Saleh et al.**

**SI Figure 1**

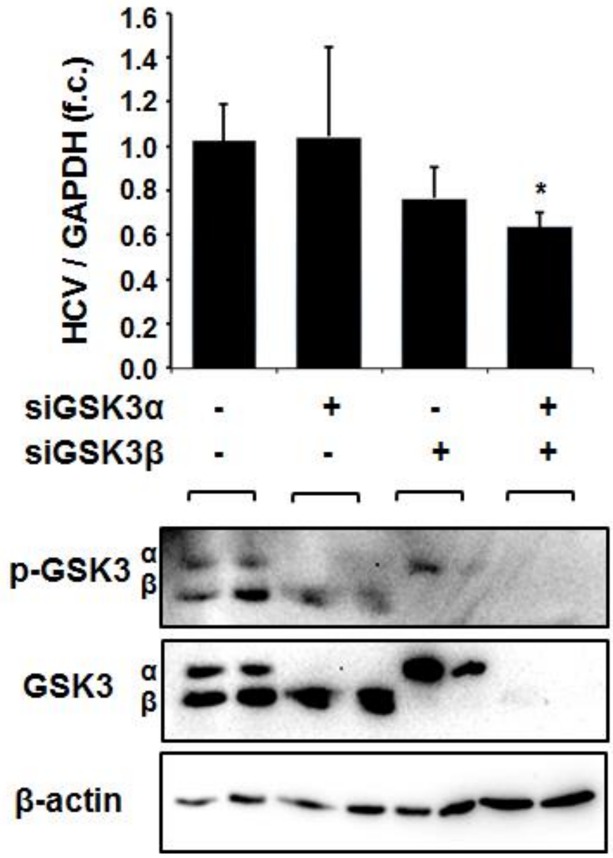


**SI Figure 1. Silencing of GSK3β suppresses HCV replication.** Huh-7.5 cells harboring subgenomic Con1 replicon were transfected with a second set of siRNAs (compared to Figure 4) against GSK3α (siRNA #6238), GSK3β (siRNA #6240) or both for 72 hours. HCV RNA levels normalized to GAPDH mRNA are expressed relative to untreated cells. Data are presented as mean ± SEM. Asterisks denote statistically significant differences (*, P-value ≤ 0.05). Immunoblot analysis was carried out for GSK3α/β to confirm gene silencing efficiency. β-actin was detected as a loading control.
